# OPM-MEG reveals dynamics of beta bursts underlying attentional processes in sensory cortex

**DOI:** 10.1038/s41598-025-08037-8

**Published:** 2025-08-19

**Authors:** Gonzalo Reina Rivero, Zoe Tanner, Lukas Rier, Ryan M. Hill, Vishal Shah, Molly Rea, Cody Doyle, James Osborne, David Bobela, Peter G. Morris, Karen J. Mullinger, Elena Boto, Niall Holmes, Matthew J. Brookes

**Affiliations:** 1 Sir Peter Mansfield Imaging Centre, School of Physics and Astronomy, University of Nottingham, University Park, Nottingham, NG7 2RD UK; 2 Cerca Magnetics Limited, 2 Castlebridge Office Village, Kirtley Drive, Nottingham, NG7 1LD UK; 3 QuSpin Inc., 331 South 104th Street, Suite 130, Louisville, CO 80027 USA; 4 Centre for Human Brain Health, School of Psychology, University of Birmingham, Birmingham, B15 2TT UK

**Keywords:** Beta oscillations, Bursts, Somatosensory, Inhibition, optically pumped magnetometers, Magnetoencephalography, MEG, Attention, Biological physics

## Abstract

Human brain electrophysiology is dominated by rhythmic activity—neural oscillations—which play an important role in coordinating brain function. In the somatosensory cortices, the dominant oscillations occur in the beta (13–30 Hz) band and are thought to mediate top-down inhibition of primary cortices. The non-invasive measurement of such oscillations has traditionally been made possible using either electroencephalography (EEG) or magnetoencephalography (MEG), yet both modalities have significant limitations. Here, we use a new MEG technology—based on optically pumped magnetometers (OPM-MEG)—to measure attentional modulation of beta oscillations in the somatosensory cortex. Using piezo-electric crystal stimulators, we present ‘braille-like’ patterns to the left and right index fingers; participants are asked to respond to pre-specified target patterns presented to an attended hand, whilst ignoring patterns presented to their non-attended hand. In agreement with previous findings, we measure significantly decreased beta amplitude during attended stimuli relative to non-attended stimuli, with the most pronounced effect immediately following an attentional cue. Moreover, we extend our analyses to demonstrate that attention has a similar effect on the occurrence of pan-spectral bursts (which underlie the beta rhythm). Specifically, we measure significant changes in the probability of burst occurrence in primary somatosensory cortices in a time window related to attentional shifts. In sum, our results provide evidence that attentional modulation of beta oscillations is driven by changes in pan-spectral burst occurrence and add weight to the argument that OPM-MEG is becoming the technique of choice for non-invasive electrophysiological measurements.

**Supplementary Information:**

The online version contains supplementary material available at 10.1038/s41598-025-08037-8.

## Introduction

Electrical activity in the human brain is dominated by rhythmic fluctuations in electrical potential across neuronal assemblies, termed ‘neural oscillations’. These rhythms play an important role in brain function, for example by coordinating spatially separate regions to promote efficient transfer of information^1^. In the sensorimotor cortices the dominant oscillations occur in the “beta” (13–30 Hz) band. The precise functional role of these oscillations remains unclear, however the amplitude of beta oscillations is robustly modulated by both sensory and motor tasks^2^. Specifically, during motor output or sensory stimulation we observe a decrease in beta amplitude, followed by a ‘rebound’ (i.e. an increase above baseline) on movement (or stimulus) cessation. This has led to theories that high beta amplitude reflects top-down inhibition of primary sensorimotor cortices (see Barone and Rossiter, 2021^3^, for a review). The importance of understanding these phenomena is underscored by studies showing that they appear abnormal in patients with a variety of neurological and psychiatric conditions; from disorders that strike in childhood (e.g. Autistic spectrum disorders^4^) to problems typically associated with older people (e.g. Parkinson’s disease^5^). Developing a better understanding of oscillatory dynamics is therefore not only critical to our understanding of brain function but could also shed significant light on how neural dysfunction affects brain health.

The “classical” view of beta oscillations is that they comprise a smooth continuous wave, whose amplitude changes during a task. However, recent studies^6–9^ suggest that this is likely an artifact of trial averaging. Briefly, when looking at unaveraged data, the beta band appears dominated by punctate ‘bursts’ of activity and the probability of burst occurrence modulates with a task^7,10^. When averaged over many trials, a summation of bursts occurring at different times gives the appearance of a smooth oscillations, whose amplitude change in time. The rate and precise timing of beta bursts have been linked to behaviour and task performance metrics, such as reaction time^7–9,11–17^. Recent studies further suggest that bursts characteristics change with age (from childhood to adulthood^18,19^) and that bursts occurring during different phases of a task (e.g. before and after a movement) have different spectral, spatial and temporal properties, suggesting different functional roles^20,21^. These insights have led to the development of biophysical models^8,9,22,23^ with evidence suggesting that variability in burst morphology can be explained by variability of synaptic inputs to neural assemblies. In sum, these recent studies have resulted in a fundamentally new understanding of beta dynamics, their modulation by tasks, and their mechanism of generation.

Measurement of beta dynamics has been performed (non-invasively) using electroencephalography (EEG)^24^ or magnetoencephalography (MEG)^25^. EEG measures changes in electrical potential difference across the scalp, generated by current flow in neuronal assemblies. MEG measures the associated fluctuations in magnetic field. Both offer a useful metric of electrophysiology, but nevertheless have limitations. For example, EEG signals are attenuated and spatially distorted by the skull, limiting sensitivity and spatial resolution. EEG also suffers from interference generated by non-neuronal sources such as muscles^26^. In MEG, the magnetic fields experience diminished distortion/attenuation due to the skull (improving spatial resolution compared to EEG)^27^. MEG is also less susceptible to non-neuronal artifacts^28–30^. However, conventional MEG systems require cryogenically cooled sensors to measure small fields from the brain. This means a thermally insulating gap must be placed between the sensors and the scalp, limiting sensor proximity and consequently sensitivity and spatial resolution. Furthermore, MEG is expensive, sensitive to subject motion and can be impractical in some cohorts. In recent years, a new type of MEG system has employed optically pumped magnetometers (OPMs) for MEG measurement^31–39^. OPMs do not require cryogenics, and can therefore be placed closer to the head (improving signal strength and spatial specificity)^32,40–42^. In addition, OPM-MEG systems are wearable—adapting to head shape^19,43,44^ and allowing movement during a scan^45–47^. In principle, OPM-MEG provides an enhanced platform for measuring beta dynamics. However, it is still a developing technology, predominantly showcased in technical demonstrations or comparisons with existing systems. There is relatively limited literature on employing OPM-MEG to address authentic neuroscientific challenges.

One striking finding using conventional MEG was the modulation of sensorimotor beta oscillations with attention. For example, a study Bauer et al.^48^ used a sensory stimulator (an array of 8 ‘pins’, each individually controllable) to apply spatially different ‘braille-like’ patterns to either the left or right index fingers. Participants were asked to attend to a specific hand (left or right) and to respond if they felt a pre-specified ‘target’ stimulus. The findings revealed that beta amplitude modulation varied depending on the attended hand; for instance, when participants attended to their left index finger, beta amplitude decreased in the right sensory cortex. The reverse was true when participants attended to their right hand. These modulations were subtle (compared to the effects of the stimulation itself), but added significant weight to an argument that beta oscillations provide top-down inhibitory influence on primary cortices. In this paper, we design a similar experiment, with two goals: First, we aim to show that these subtle beta modulations with attention are measurable using OPM-MEG. Second, we aim to discover whether this attentional modulation in beta amplitude can be explained using the burst model of oscillatory dynamics.

## Materials and methods

### Tactile attention paradigm

All participants gave written informed consent to take part in the experiment, which was approved by the University of Nottingham Faculty of Health Science Research Ethics Committee. The scanning procedure was performed following relevant guidelines for OPM-MEG. Informed consent and authorisation for publication of Fig. 2B were also obtained.

The experimental paradigm^48^ involved tactile stimulation of the subject’s index fingers using braille stimulators (Metec, Germany). Each stimulator houses 8 pins arranged in a 4 × 2 matrix; each pin is controlled individually by a piezoelectric crystal which places them either up (stimulating) or down (not stimulating) when a voltage is applied. To control the voltage, we used an in-house developed electronics unit comprising a shift register and DC converter. This receives serial commands sent from a computer and translates these to a desired pattern of up and down pins on the braille cells. In this way, it is possible to present any spatial pattern of pins to the subject. The braille cells were controlled using the PsychoPy library^49^. All code used to control the task is available on GitHub (https://github.com/GonReina/Braille_paradigm.git).

**Fig. 1:**
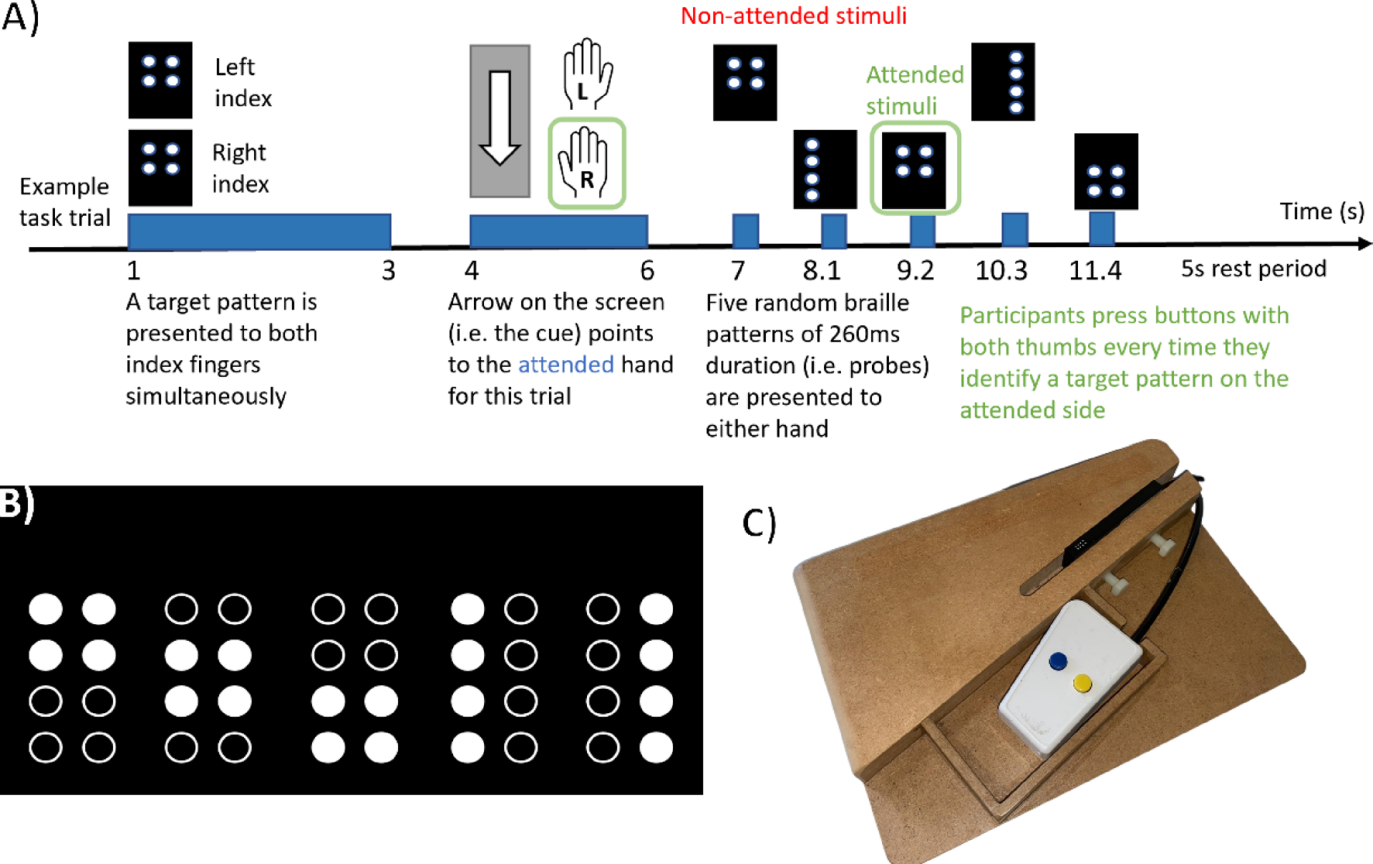
Tactile attention task. (**A**) Schematic diagram of the braille attention task for a single trial. (**B**) The five different braille patterns used during the experiment: all patterns have 4 pins up (white dots) and 4 pins down (black dots). (**C**) Wooden mount used to hold the braille stimulators and response buttons. The hand is placed such that the index finger is over the braille pins, and the blue button can be accessed by the thumb.

A schematic of the experiment is shown in Fig. 1A. A single trial lasts 16 s. In the *1 s < t < 3 s* time window, a target pattern is presented to both left and right index fingers. Following this, in the *4 s < t < 6 s* window a visual cue (an arrow pointing either left or right) is shown on a screen; this directs the subject to the attended hand. Although the order of the attentional cues (across trials) was random, half of the trials directed subjects to their left hand, and the other half directed them to their right hand. Following the attentional cue, 5 ‘probe stimuli’ are then applied pseudo-randomly to either the left or right fingers (the first at *t* = 7 s, and then at intervals of 1.1 s; each stimulus lasts 260 ms). Participants were instructed to respond via a MEG compatible response box (Current designs Inc., Philadelphia, PA) by pressing a button with both thumbs (regardless of which hand the probe was felt on) if a probe stimulus matched the target pattern on the attended hand. To simplify the experiment, we limited ourselves to 5 spatial patterns, which are shown in Fig. 1B. The target pattern was picked at random at the start of each trial (i.e. in any one trial, the chances that a specific spatial pattern would be the target was 20%). The number of target patterns presented to the attended hand per trial was drawn from a gamma distribution with a shape of 4 and a scaling factor of 0.3. The stimulators and response buttons were held in place by custom-made wooden supports (Fig. 1C). Signals were sent from the computer controlling the braille stimulators to the OPM-MEG acquisition system to delineate the timing of each event in the paradigm (henceforth termed triggers). 80 trials were recorded, along with a rest trial every 10 trials, showing feedback to the participants on their performance. Performance was calculated by taking the number of correct button presses as a fraction of the total number of presses. The total experimental length was 1408 s.

### OPM-MEG system overview

**Fig. 2:**
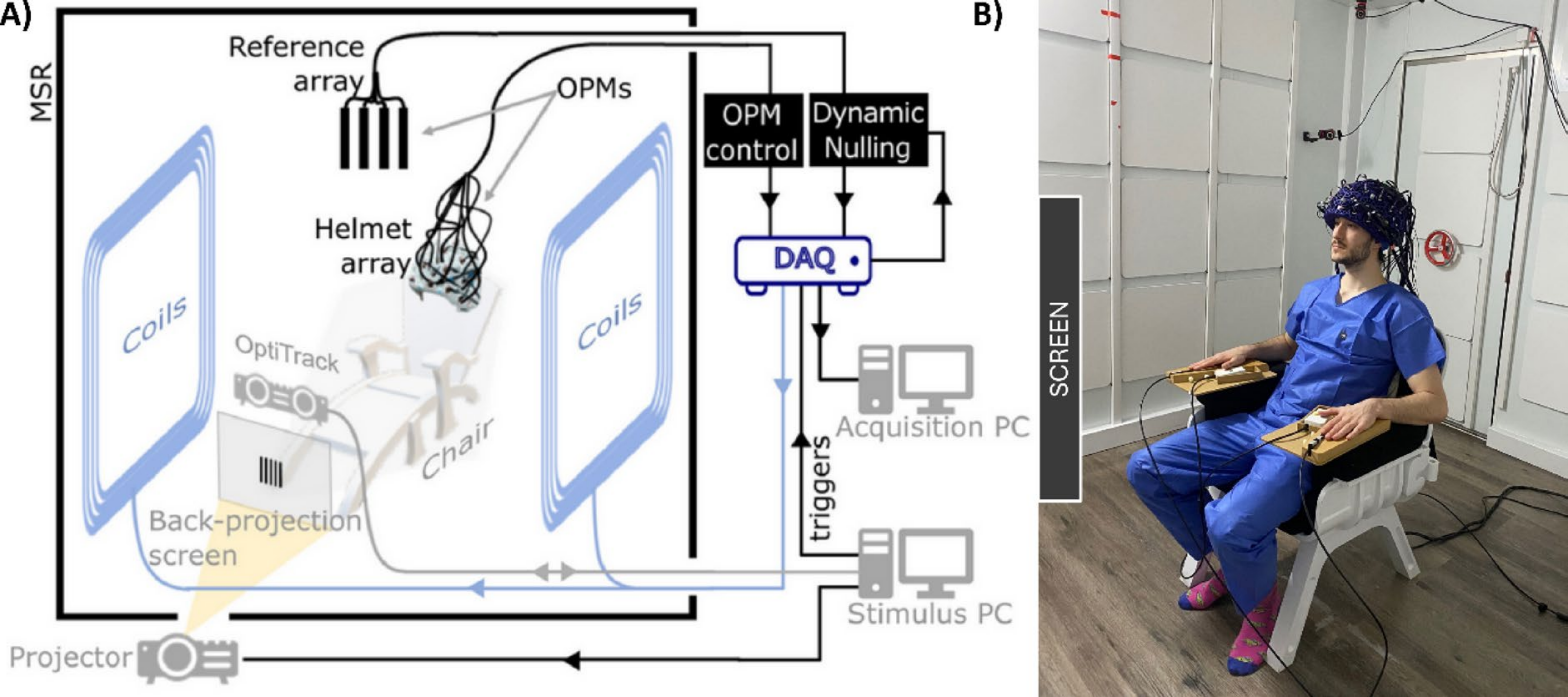
OPM-MEG system. (**A**) Schematic of the OPM-MEG system (Figure adapted from Hill et al., 2022). (**B**) Photograph of a subject completing the task (consent and authorisation for publication was obtained).

All data were collected using an OPM-MEG system at the University of Nottingham^37,50,51^ (see Fig. 2A). Participants sat in front of the screen, at the centre of a magnetically shielded room (MuRoom, Magnetic Shields Ltd., Kent, UK) with their hands resting on the wooden supports (Fig. 2B). The non-magnetic chair was placed at the isocentre of a set of bi-planar electromagnetic coils, which were used to control background magnetic fields (Cerca Magnetics Ltd., Nottingham, UK). MEG data were collected using an array of 3rd generation, triaxial, zero-field OPMs (manufactured by QuSpin Inc. Louisville, CO, USA). These were mounted in 3D-printed helmets of varying size (Cerca Magnetics Ltd.) and gave approximate whole head coverage (Fig. 2B). Background magnetic fields were measured by a second OPM array, comprising 4 dual axis OPMs (QuSpin, 1st generation) placed behind the subject. This reference array enabled feedback to the bi-planar coils to dynamically compensate any drifts in background field measured by the reference array (see also below).

Each OPM sensor head is a self-contained unit connected to an electronics control box, which powers the sensor, enables control of on-board components, and outputs an analogue signal which (in a low (< 1 nT) background field) is directly proportional to magnetic field. The OPMs were synchronised as a single array and the outputs from all sensors were connected to a National Instruments (NI, Austin, Texas, USA) Data Acquisition Unit (DAQ) (NI-cDAQ-9179) which captured the signals from all channels, alongside the stimulus triggers, at a sample rate of 1200 Hz with 16-bit precision. The OPMs were controlled by a single acquisition PC using serial commands via the (manufacturer provided) OPM control software; this was used to set up and calibrate the OPMs at the start of every recording. The same (acquisition) PC was also used to control the bi-planar coils to compensate background fields. A separate (stimulus) PC was used to control the experimental paradigm. Visual stimuli were presented via back-projection through a waveguide in the MSR and onto a screen located ~ 1 m in front of the subject. (We used a ViewSonic PX748-4 K projector). A system of 6 motion tracking cameras (OptiTrack Flex 13, NaturalPoint Inc., Corvallis, Oregon, USA) was also available to track motion of infra-red retroreflective markers, which were attached to the helmet. This allowed real-time head motion tracking.

As a consequence of using a novel system, the setup changed over time, being subject to maintenance, upgrades and alterations. This meant that some subjects were scanned with a different number of channels in the array. Initially, 9 subjects were scanned using a 132-channel array. A second set of scans, in 15 subjects (6 of whom had been scanned previously) was then performed using a 192-channel array. This yielded a total of 24 datasets from 18 participants. Appendix 1 shows an investigation into the impact of the variation in channel counts on our analysis, which was found to be minimal in the brain regions of interest. To avoid conflation of inter- and intra-subject variance, in our final analyses only one scan per subject (that with the higher channel count) was used.

### Background magnetic field control

OPMs require low (< 1 nT) background magnetic fields to ensure a linear output (see e.g. Schofield et al., 2023^52^ for a review). Here, the magnetic field inside the MSR was controlled using a 3-step process, carried out for every scan. Firstly, the inner mu-metal walls of the MSR were demagnetised using a degaussing procedure^53^, resulting in a drop in magnetic field magnitude from ~ 8 nT to ~ 2 nT. Following this, dynamic field compensation was applied, using the reference array to measure change in background field over time and the bi-planar coil to compensate for any measured drift. Once enabled, this ensured a temporally stable (but non-zero) background field. Finally, we used a field mapping and compensation technique^54,55^ to remove the remaining static field. Briefly, participants performed a series of head movements during which changes in magnetic field at the OPMs were recorded alongside synchronised head motion data. These data were then fitted to a spherical harmonic model of magnetic fields across the volume occupied by the helmet, to estimate the strength and spatial variation of the field. An equal and opposite (DC) field was then generated by the bi-planar coils. This nulling process reduces remnant static background magnetic field to ~ 0.3 nT and ensures accurate data are recorded with minimal motion artefact^51^.

### Co-registration

MEG data can be mathematically modelled to create 3D volumetric images, showing moment-to-moment changes in brain electrophysiology. This process—termed source-space projection—requires accurate knowledge of the OPM locations and orientations with respect to brain anatomy. To achieve this, we used an optical technique based on structured light^33,56^. Immediately prior to MEG data acquisition, two 3-dimensional optical scans of the subject’s head were acquired. In the first scan, the subject was asked to wear a swimming cap (to flatten their hair); this was used to get an approximation to head shape. In the second scan, the subject was scanned with the OPM-MEG helmet in position; this measured the location of the helmet relative to the face. In both cases data were obtained using either a Skanect (Version 1.11.0, Occipital Inc., San Fransisco, CA, USA, support.structure.io) infrared structured light scanner (for the first set of 9 subjects) or a 3D structured light scan (Einscan H, Version 1.2.1.1, SHINING 3D, Hangzhou, China, support.einscan.com) (for the second set of 15 subjects). The resulting 3D meshes of the subject’s face/head were aligned with each other using Meshlab^57^ (MeshLab-022.02, http://www.meshlab.net). The scan with the helmet was combined with knowledge of sensor geometry inside the helmet (from the helmet 3D-printing process) to delineate the sensor locations and orientations relative to the face and scalp. Finally, every subject had previously undergone a T_1_ weighted anatomical MRI scan (3T Phillips Ingenia; MPRAGE sequence; 1 mm isotropic resolution). By extracting the scalp surface from the MRI and fitting it to the optical digitisation of the head (without the helet), we enabled complete co-registration of the sensor locations/orientations to brain anatomy.

### Data pre-processing

All datasets (including co-registration information) were organised according to the Brain Imaging Data Structure (BIDS) guidelines^58^. Data pre-processing was carried out using a pipeline developed in-house and implemented in MATLAB (Version R2023a, MathWorks Inc.). First, power spectral density (PSD) measures for all channels were generated using Welch’s method^59,60^. This approach involves dividing the recording into segments and then averaging adjusted periodograms of these segments. PSD results were inspected to ensure there were no major artifacts in the data. This led to the removal of two datasets: the first due to noisy signals caused by a power supply fault, and the second due to missing data caused by a problem with the acquisition software. This left a total of 22 usable datasets from 16 participants. (Of the remaining subjects, 14 were right-handed; 8 identified as male, 8 female; the average subject age was 27.4 ± 3.2.) Bad channels were visually identified by plotting both the channel time course and the PSD (using MNE-python,, Version 1.6.0^59^). Any channels with excess noise (above ~ 16 fT/sqrt(Hz) in the 5–150 Hz range) or a PSD of zero were removed. Following this, data were segmented into trials. To eliminate bad trials, a standard deviation threshold was identified by combining data across all trials, calculating the mean and standard deviation, and then computing the standard deviation values for each trial individually. Any individual trial (including rest blocks) with a standard deviation value exceeding three times that of the combined data was removed. Following this, homogeneous field correction was performed to attenuate the effects of interference that manifests as spatially uniform magnetic field changes across the sensor array (as such uniform fields will not represent brain activity)^61^.

### Source space analysis

We chose to carry out our analysis in source space for three reasons: (1) It allows us to target the sensorimotor cortex, which is known to generate beta oscillations^3,62^. (When analyses are carried out at the sensor level, the mixing of signals from different brain regions^63^ means we cannot confidently attribute observed beta dynamics to specific cortical generators.) Second, the signal-to-noise ratio of MEG data is improved substantially through source localisation^64,65^. This improvement in SNR is helpful for accurate characterisation of brain dynamics, particularly when—as in this paper, we sought to measure relatively subtle (attentional) effects. Third, previous literature on sensorimotor beta activity has used similar approaches; this methodological consistency enables us to contextualize our findings within the established literature.

To achieve source localisation, the brain was first parcellated into 78 cortical regions, defined by the automated anatomical labelling (AAL) atlas^66^. This was achieved by warping a template brain to each individual’s anatomy using FSL’s FLIRT tool^67,68^ (FSL-6.0.6.2, fsl.fmrib.ox.ac.uk) and applying the same transform to the AAL atlas. Source positions were located at the centre of mass of each AAL region and sensor signals projected to each source location using a linearly constrained minimum variance (LCMV) beamformer^63^. Forward solutions were calculated based on a current dipole and a single shell volume conductor model^69^ using Fieldtrip^70^ (Fieldtrip-20221223, http://www.fieldtriptoolbox.org/download). Covariance matrices were computed separately for distinct frequency bands of interest, including broad-band (1–48 Hz) and beta-band (13–30 Hz). These matrices were regularised using the Tikhonov method with a regularisation parameter equal to 5% of the maximum eigenvalue of the unregularized covariance matrix^64^. Beamformer reconstructions of the timecourse of electrophysiological activity (termed virtual electrodes) were constructed for each region, and separately in each frequency band, with source orientation taken as that with the largest signal. The result was both a broadband and beta band virtual electrode timecourse for each of the 78 brain regions, in each of the 16 subjects.

### Beta envelopes

For each brain region, we estimated instantaneous task induced contrast to noise ratio (CNR) in the beta band as a measure of change in beta amplitude. Specifically, beta filtered data from the whole experiment were Hilbert transformed to generate the analytic signal; the absolute value of this provided the ‘Hilbert envelope’ which shows the instantaneous oscillatory amplitude as a function of time. Following this, two separate analyses were carried out relative to both the 16 s *trials* and the individual *probe stimuli*:

- Trials: Data were segmented into 16-s trials, which were split into two groups—attend left and attend right. Baseline mean and standard deviation were estimated based on the last 8 s of the interspersed rest trials (I.e. baseline was recorded in a window that started at least 15 s after the last stimulus was presented). Envelope data for all trials were averaged within each group, baseline corrected (by subtracting the mean beta amplitude in the rest trials) and normalised (by dividing by the standard deviation of the rest trials). This resulted in an instantaneous CNR timecourse reflecting the change in beta envelope from baseline as a percentage of standard deviation of the baseline signal.
- Probe stimuli: Data were also segmented into time windows from − 180 ms to + 650 ms relative to the probe stimuli. There were 4 categories of probe: attend left stimulate left; attend left stimulate right; attend right stimulate left; attend right stimulate right). We averaged envelope signals for each type of probe and again baseline corrected and normalised (using the rest trials). We removed target stimuli (which only occur for attended stimuli) from the analysis, and this led to having more non-attended than attended trials. This difference was addressed by removing randomly selected probes to equalise the trial counts.

All calculations were performed separately for every subject, and data were averaged across subjects.

### Burst analysis

We used a univariate time delay embedded Hidden Markov Model (HMM), applied to the 1–48 Hz virtual electrode data, to identify bursts. This method has been described extensively in previous papers^10,71–73^; a more detailed mathematical description can also be found^74,75^.

Briefly, we took the virtual electrode time series and used the HMM to identify the occurrence of three “states” each depicting repeating patterns of activity with similar temporo-spectral signatures. The output was three timecourses representing the likelihood of each state being active as a function of time. These were binarized (using a threshold of 2/3) to provide a timecourse of state occurrence. Note that the Markovian property means that the likelihood of state occurrence is dependent only on the previous state; states are mutually exclusive and all time points in the virtual electrode timecourse must belong to one of the three states. The state whose timecourse correlated highest with the beta envelope was defined as the “burst state”.

The binarised burst timecourses were taken for each trial and, for each time point within a trial, we measured the statistical likelihood of burst occurrence. This was computed as the number of trials with a burst at that time point, divided by the total number of trials. Timecourses of burst probability were constructed for both trial types (attend left and attend right). In addition, we measured burst probability (in the same way) for the four probe types (attend left stimulate left; attend left stimulate right; attend right stimulate left; attend right stimulate right). Unfortunately, one subject was excluded from the analyses due to an inadequate fit by the HMM.

In theory, a burst of activity need not be restricted to a single frequency band (e.g. hypothetically, a burst might resemble a ‘chirp’ which would encompass many frequencies). For this reason, in addition to detecting bursts, we also sought to determine their spectral content and the use of broadband-filtered data and time embedding enables this. Specifically, we used a state-wise multi-taper analysis method, developed in existing work^76^ to derive a spectrum for each of the three states, showing the frequency content of the MEG signal during each state occurrence. This approach presents advantages over more traditional approaches (which usually involve spectral filtering to the beta band and thresholding beta amplitude) since it eliminates the underlying assumption that bursts are exclusively generated by the beta band.

### Statistical analyses

Our primary hypothesis was that, using OPM-MEG, we would observe attentional modulation of beta amplitude. To this end, we undertook three sets of statistical analyses.

1. Beta modulation by attentional cue: We averaged beta amplitude across the time window when the attentional cue was presented (i.e. 4 s < t < 6 s relative to trial start). We then conducted two statistical tests. First, looking in left sensory cortex (where we expect the largest activity for right-hand stimulation), we contrasted right-cued (attended) versus left-cued (non-attended) trials. We predicted lower beta amplitude following an attended cue. Second, looking in right sensory cortex (largest activity for left-hand stimulation) we again contrasted right-cued (non-attended) versus left-cued (attended) trials, predicting lower beta amplitude following an attended cue. In all cases, beta amplitudes were averaged over trials for each subject, and statistical significance was assessed across subjects using a non-parametric Wilcoxon sign-rank test.
2. Beta modulation during probe stimuli. Beta amplitude was averaged in the 0 ms < t < 260 ms window relative to the presentation of probe stimuli. (All target stimuli, or stimuli where a button was incorrectly pressed, were excluded from these analyses to remove confounds of the button press and the number of attended/non-attended stimuli were equalised as detailed above.) We conducted two statistical tests. First, we looked in left sensory cortex following probe stimuli presented to the right hand, and contrasted beta amplitude when subjects were attending to their left (non-attended) or right (attended) hand. We expected smaller beta amplitude when the right hand was attended. Second, we looked in right sensory cortex following a left-handed probe and again contrasted beta amplitude with attention. Here, we expected smaller beta amplitude when the lefthand was attended. Significance was assessed using a Wilcoxon sign-rank test.

In total, for the above analyses we conducted 4 statistical tests. Multiple comparisons were controlled using the Benjamini-Hochberg method and we set the false discovery rate to 5%.

Our second hypothesis was that any measurable effect of attention on beta amplitude would be mirrored by a change in the dynamics of pan-spectral bursts. To test this, we conducted the same tests as those described above, but using burst probability timecourses derived using the HMM (rather than beta envelope). This allowed us to test whether there were significant changes in burst probability with attentional shift. However, a change in burst probability could be driven by either an increase in the number of bursts per unit time (burst count) or by a change in burst duration. Similarly, a change in beta envelope could result from altered burst count, duration, or amplitude. We therefore derived three additional burst-related metrics:

1. Burst count: The number of bursts occurring within a time window. Note, bursts were only counted if they were fully encapsulated (i.e. started and ended) within the window of interest.
2. Burst duration: Defined as the length of time (for a single burst) that the binary time series delineating the burst state was set to 1 by the HMM binarization.
3. Burst amplitude: We took the 1–48 Hz timeseries (on which the HMM was based), computed the Hilbert envelope (as above) and found the maximum amplitude during each burst.

These three measurements were made on all bursts occurring during presentation of the attentional cues (4 s < t < 6 s relative to trial start) and during the window around probe stimuli (0 s < t < 1.1 s relative to the probe onset). In the former case we contrasted left and right attentional cues and measured the difference in count, amplitude and duration. In the latter case we contrasted attended and non-attended probes (and measured the same differences). In all cases, measures were derived in left and right somatosensory cortices independently and statistical significance was assessed using a Wilcoxon sign-rank test. This resulted in 12 separate statistical tests (3 metrics × 2 windows × 2 brain regions). Multiple comparisons were again controlled using the Benjamini–Hochberg method.

## Results

### Task performance

In general subjects found the task difficult. The overall accuracy (defined as the number of correct button responses (i.e. button presses following a target) divided by the total number of times a subject made a button press) was 51.25 ± 20.51% (mean ± standard deviation over all subjects). Accuracy was also calculated independently for left-cued and right cued trials, but we found no significant difference between them. Accuracy was also measured in the first third, and last third of trials, to test for a learning effect, however we again found no significant difference.

### Beta band amplitude modulates with attention

**Fig. 3:**
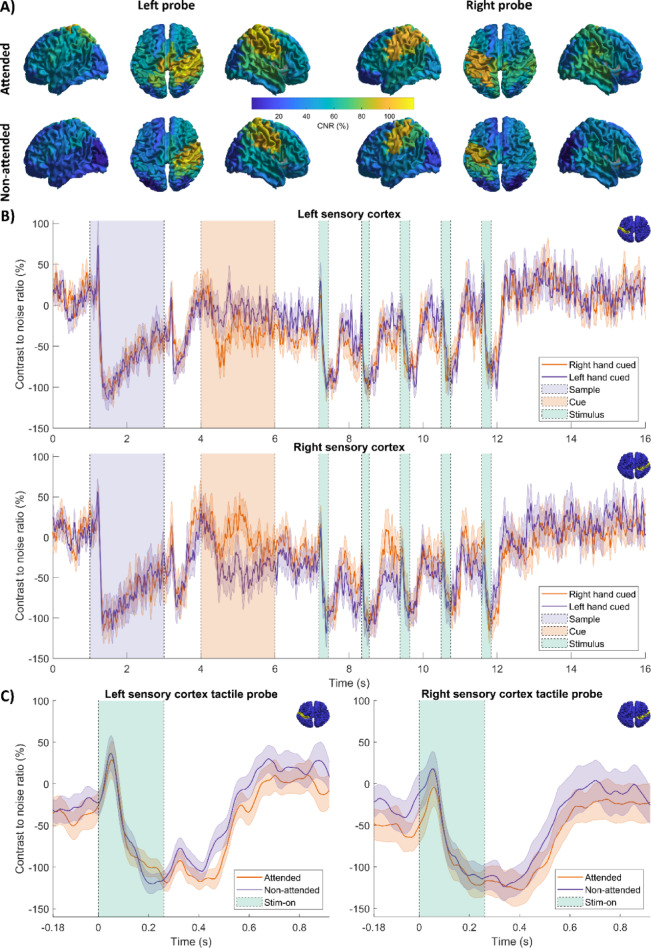
Beta CNR changes with attention: (**A**) Colourmaps show beta CNR measured 300 ms after the onset of the probe stimulus. This point in time was selected as the largest deflection in the beta envelope from baseline on average between conditions in the somatosensory cortices. The top left panel shows attend left, left hand probe; top right panel is attend right, right hand probe; bottom left panel is attend right, left hand probe and bottom right panel is attend left, right hand probe. The brain surface colourmaps were produced using the AAL atlas brain template in MATLAB (Version R2023a, MathWorks Inc.). (**B**) Beta CNR timecourses for the 16 s trial. Panel i shows left somatosensory cortex; panel ii shows right somatosensory cortex. In both cases the orange and purple traces show trial averaged beta CNR for right and left cued trials, respectively. The shaded areas show standard error on the mean across subjects. (**C**) Beta CNR timecourses following presentation of a probe stimulus. (i) Left Somatosensory cortex. (ii) Right somatosensory cortex. Here the orange and purple timecourses represent attended and non-attended probes.

Figure 3A shows the spatial signature of instantaneous beta band envelope (CNR), at a time point ~ 300 ms following presentation of a probe stimulus. (This is the median time at which CNR showed the largest difference from baseline across all conditions). The left and right sub-panels show stimulation to the left and right hands, respectively. The upper- and lower-sub-panels show attended and non-attended stimuli. As expected, the postcentral gyri (primary sensory cortices) exhibited the highest CNR, with left hemisphere dominant for probes presented to the right hand, and vice versa. There were no measurable spatial differences (at the scale of the AAL brain parcellation) for attended and non-attended stimuli.

Figure 3B shows beta envelope timecourses throughout a trial. The upper plot shows the envelope for the left somatosensory cortex and the lower plot shows the equivalent for the right somatosensory cortex. In both cases, the orange trace shows right-cued trials and the purple trace shows left-cued trials. The shaded areas depict standard error on the mean across subjects. The data show clearly that sensory stimulation produced a marked drop (of around 100%, relative to the standard deviation of the resting signal, measured over time) in beta amplitude relative to baseline, as expected; this is clear for both the target stimulus (shown by the purple shaded area) and probe stimuli (shown by the green shaded areas).

Throughout the start of a trial, the presentation of the target stimuli, and towards the end of the trial, beta envelopes for left- and right-cued trials are very similar (no effect of attention). However immediately following presentation of the attentional cue, and during the probe stimuli, they appear to diverge. Statistical analysis showed that for both hemispheres, there was a significant difference following presentation of the attentional cue (the 4–6 s window) suggesting beta envelope modulation by attention. Specifically, in left sensory cortex, right cued trials (i.e. attention directed to the relevant hand) resulted in significantly lower beta envelope following the cue (*p* = 0.0053*; Wilcoxon sign-rank test—*indicates the effect remains significant following multiple comparison correction)). Similarly, in right sensory cortex, left cued trials resulted in significantly lower beta amplitude (*p* = 0.00006*; Wilcoxon sign-rank test). This confirms the hypothesis that, using OPM-MEG, we were able to measure attentional modulation of beta amplitude – similar to that detected previously in conventional MEG^48^.

A post-hoc analysis on the sensory cue (which was not part of our initial hypothesis) also showed that, in the 4.5–6 s time window, in the left sensory cortex with a left cue, beta amplitude wasn’t significantly different to baseline (i.e. not shifted from zero in the plot) (*p* = 0.72; Wilcoxon Sign rank test) whereas with a right cue the beta amplitude decreased from baseline (*p* = 0.0067; Wilcoxon Sign rank test). Similarly in the right hemisphere, with a right cue beta amplitude wasn’t significantly different to baseline (*p* = 0.60; Wilcoxon Sign rank test) whereas with a left cue the beta amplitude decreased from baseline (*p* = 0.0067; Wilcoxon Sign rank test). In other words, even in the absence of tactile stimulation a sensory cue produces a significant drop in beta amplitude in sensory cortex, if the cue points to the contralateral hand; a cue to the ipsilateral hand produces no significant effect.

Figure 3C shows the beta envelope following probe stimuli. The left-hand plot shows data for the left somatosensory cortex and the right-hand plot shows the right somatosensory cortex. In both cases, the orange trace shows attended and the purple trace non-attended stimuli; again, the shaded area represents standard error over subjects. The beta amplitude during presentation of attended stimuli is lower throughout the whole window (0 s to + 1.1 s relative to the probe presentation; *p* = 0.018* in left sensory cortex and *p* = 0.0215* in right sensory cortex). Post hoc analysis of separate time windows during and after stimulation showed that beta envelopes have approximately the same amplitude during stimulation (0 ms to 260 ms; *p* = 0.49 for left sensory cortex *p* = 0.022 for right sensory cortex). With a significant decrease in amplitude for attended cases in a time window following stimulus offset—though this was only in the left sensory cortex (260 ms to 840 ms; *p* = 0.0084).

### Pan-spectral burst probability modulates with attention

**Fig. 4:**
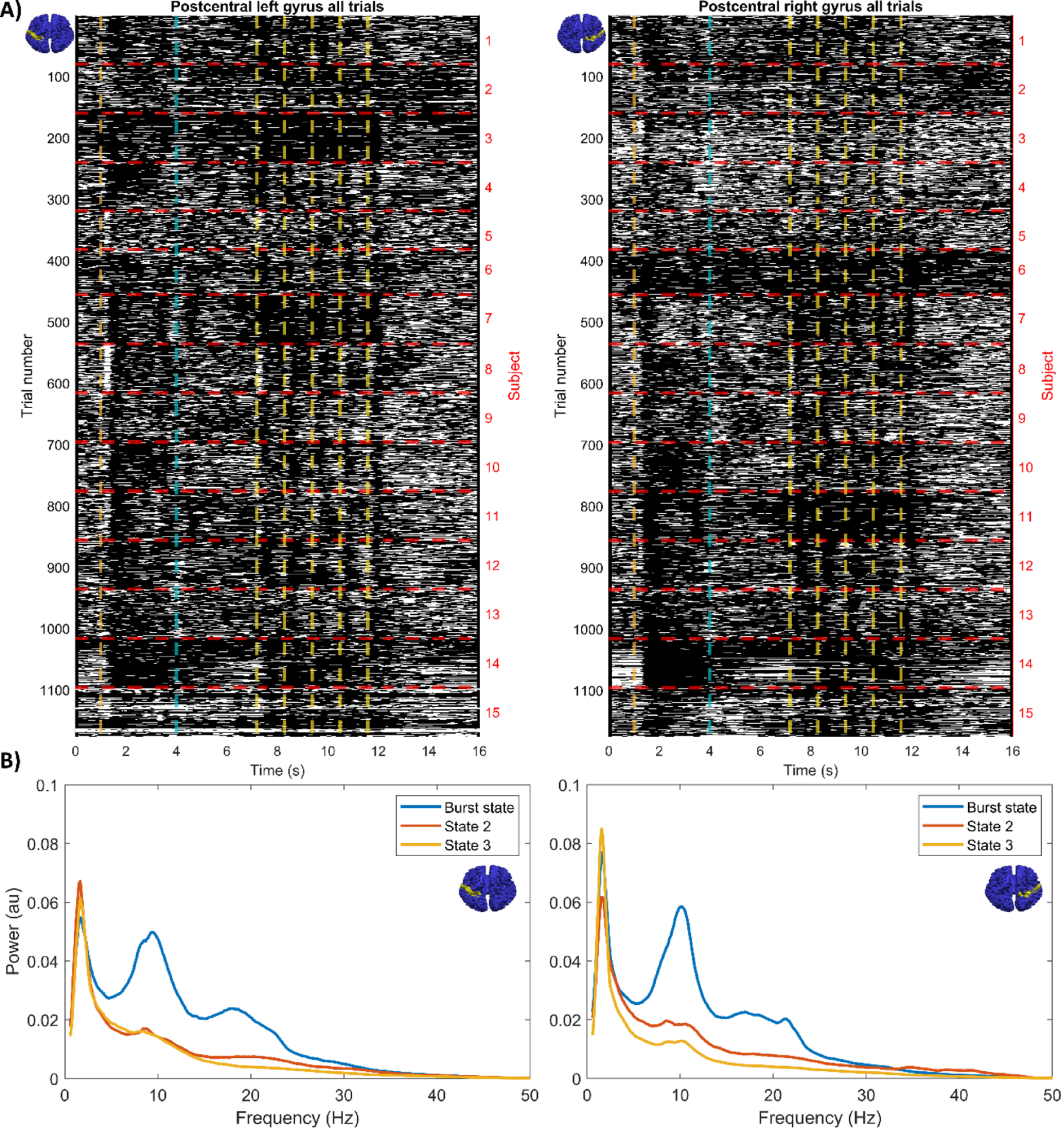
Pan spectral bursts during the task: (**A**) Raster plots showing the binary timecourse of the burst state for all trials. Trials are concatenated in the y-axis. The x-axis represents time. White indicates presence of a burst, whereas black delineates the absence of a burst. Left panel shows the left somatosensory cortex, and right panel shows the right. (**B**) Power spectral density plots for the three HMM states; the blue trace shows the burst state. Again, left and right panels respectively correspond to the left and right somatosensory cortices as indicated by the small brain colourmaps on each corner. These were produced using the AAL atlas brain template in MATLAB (Version R2023a, MathWorks Inc.).

Figure 4A shows a raster-plot of burst occurrence in individual experimental trials. Panel i shows data for left sensory cortex, and panel ii shows right sensory cortex. In both cases, white indicates occurrence of a burst; black the absence of a burst. All trials for all subjects are concatenated in the y-axis. The onset of the target pattern, attentional cue and probe stimuli are shown with the dashed vertical lines; dashed horizontal lines delineate subjects. Note that the task structure can be seen clearly in these single trial data, with bursts less likely following sensory stimulation (both the target and the probes). It is also noteworthy that the burst structure changes markedly between subjects. Figure 4B shows power spectral density for each of the three states found by the HMM. Left and right somatosensory cortices are shown in the left and right plots respectively, and in both cases the burst state is shown in blue. Notice the pan-spectral nature of the bursts, with components not only in beta band but also at lower frequencies, including a dominant peak in the alpha band. This will be addressed further in our discussion.

**Fig. 5:**
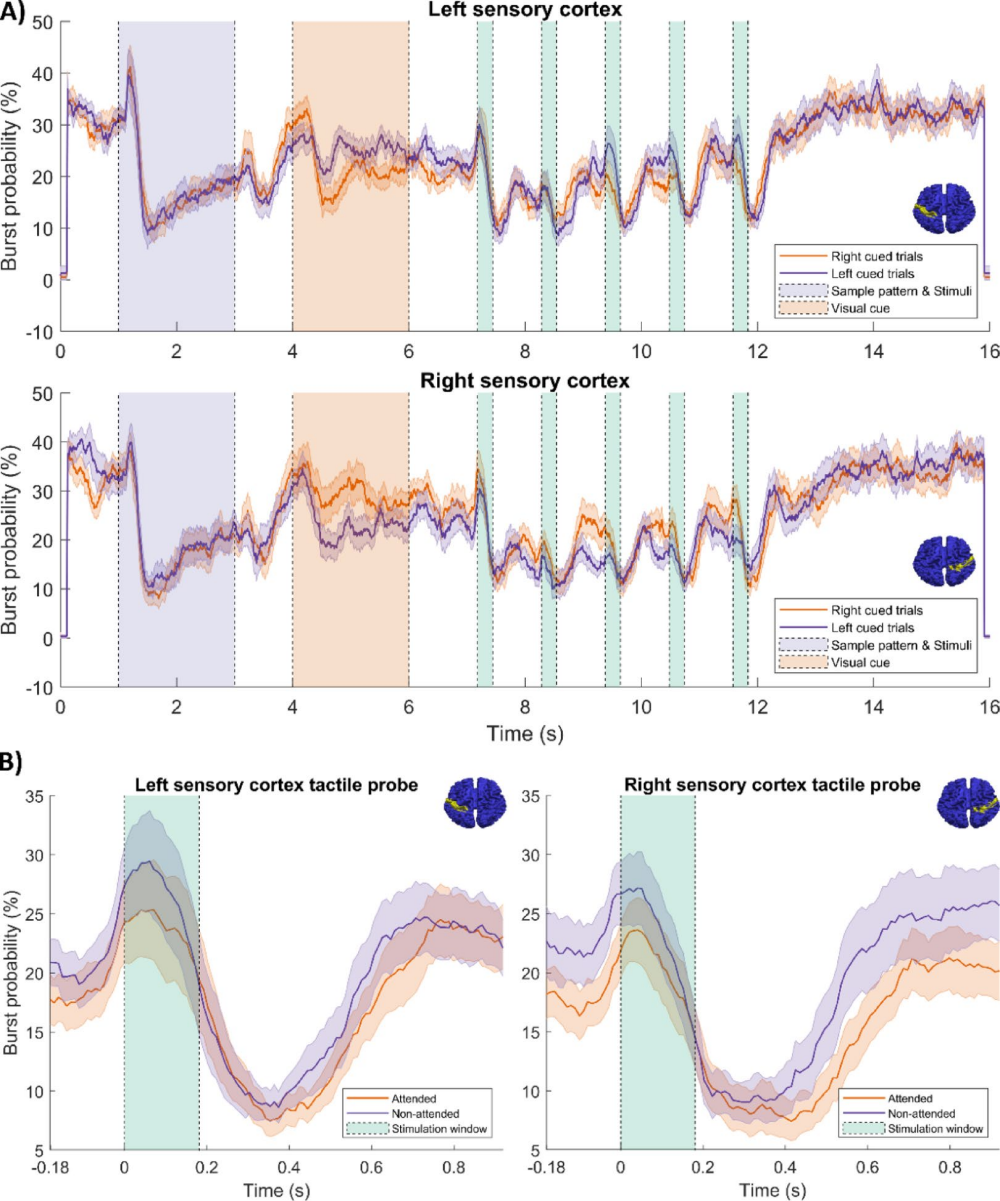
Modulation of pan-spectral burst probabilities by the task: (**A**) Burst probability for the whole trial. Panel i shows left somatosensory cortex; panel ii shows right somatosensory cortex. The orange and purple traces show burst probability for right and left cued trials, respectively. The shaded areas show standard error across subjects. (**B**) Burst probability following presentation of a probe stimulus. (i) Right Somatosensory cortex. (ii) Left somatosensory cortex. Orange and purple timecourses represent attended and unattended probes. The brain surface colourmaps were produced using the AAL atlas brain template in MATLAB (Version R2023a, MathWorks Inc.).

Figure 5 shows how the probability of bursts modulates throughout the task. Changes in burst probability during whole trials are shown in Fig. 5A. The upper plot shows left somatosensory cortex and the lower plot shows the right somatosensory cortex. The orange traces show right-cued trials and the purple traces show left-cued trials. Sensory stimulation produced a drop (of ~ 20%) in burst probability. As with the beta envelope, throughout the start/end of a trial and the presentation of the target stimuli, burst probability for left- and right-cued trials is similar, but the two diverge following presentation of the attentional cue; this is true for both hemispheres. Specifically, there was a significant difference between left and right cue in the 4 s to 6 s time window: (in left hemisphere; *p* = 0.0083*; in right hemisphere *p* = 0.0006*). Figure 5B shows burst probability following probe stimuli. The left and right plots show results for the left and right somatosensory cortices respectively; for both, orange shows attended and purple non-attended stimuli. In both hemispheres, post-stimulus burst probability was slightly higher in the non-attended case, but this only reached significance in the right hemisphere (time window 260 ms to 840 ms; *p* = 0.0181*).

**Fig. 6:**
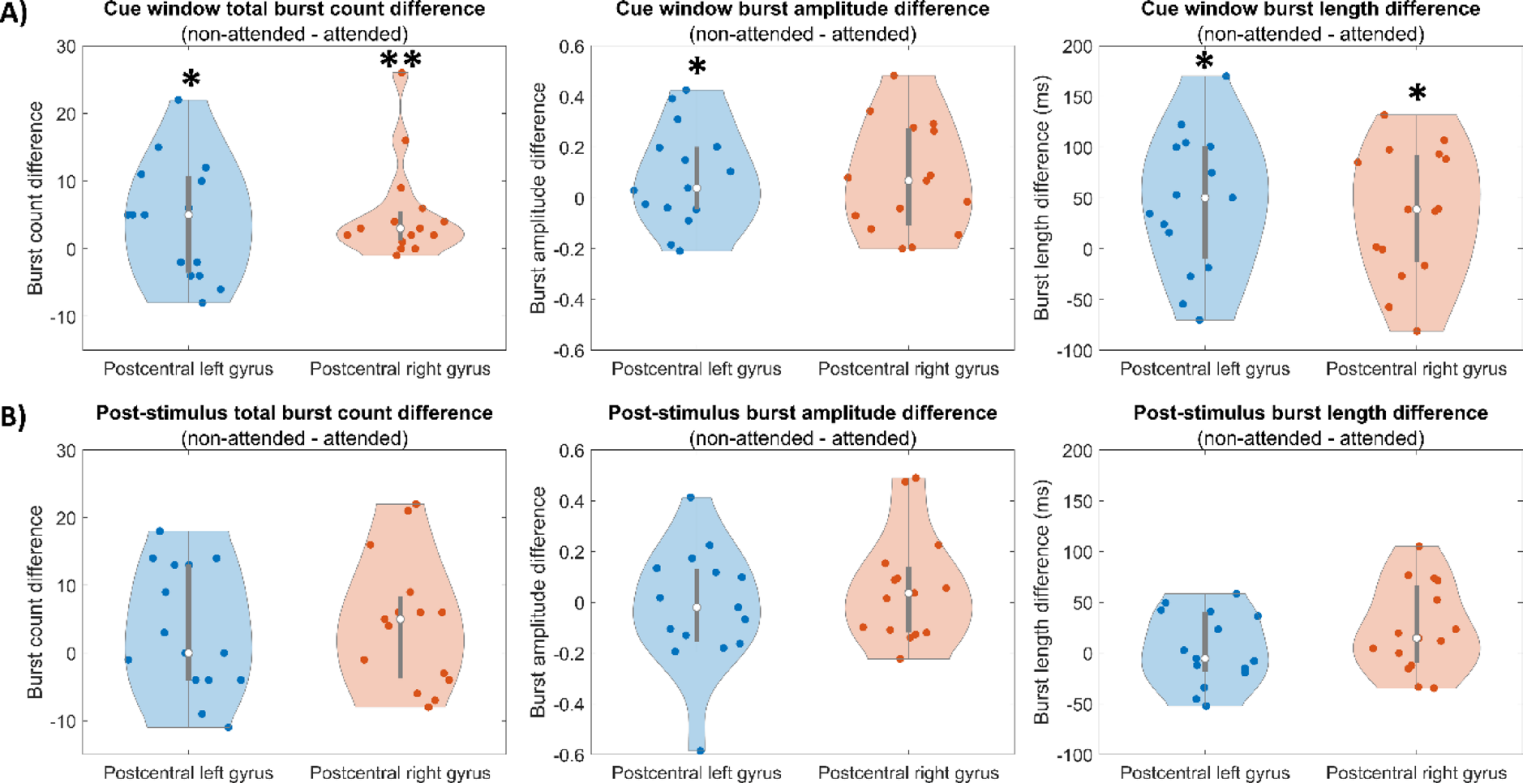
Burst statistics: The left, centre and right column show measurements of attentional differences in burst count, burst amplitude, and burst duration, respectively. In all cases, (**A**) shows the difference between left and right attentional cues (bursts in the 0 s to 2 s window relative to the onset of an attentional cue). (**B**) Shows attended and non-attended probes (bursts in the 260 ms to 840 ms window relative to presentation of a probe). In all cases, data for the left (blue) and right (red) hemispheres are shown. Measurements show a significant effect of attention if they differ significantly from zero (**denotes significance following multiple comparison correction; *denotes a p-value < 0.05 uncorrected).

Finally, Fig. 6 shows how burst count, amplitude and duration are modulated by attention. In the top row, we plot the difference between left and right attentional cues in the 0 s to 2 s window following cue onset. Note that in the plots, a “meaningful” attentional effect occurs for a pattern of results that differs significantly from zero. In right sensory cortex, burst count was significantly higher following a right (non-attended) cue compared to a left (attended) cue (*p* = 0.0007). There was also a trend towards a longer burst length (*p* = 0.048) during the cue, though this didn’t survive multiple comparison correction. In left sensory cortex there was a trend showing higher burst count following a left (non-attended) compared to right (attended) cue (*p* = 0.04). There were also trends towards higher burst amplitude (*p* = 0.025) and longer duration (*p* = 0.012) following non-attended cues. However, none of these effects were significant following multiple comparison correction. In the time windows around the probe stimuli we saw no significant effects.

## Discussion

OPM-MEG has significant potential as a research and clinical tool. It offers a possible replacement for conventional MEG and an alternative to EEG. Nevertheless, OPM-MEG remains new technology and its adoption relies critically on demonstrations of utility within an array of neuroscientific and clinical use cases. Here, we have shown that OPM-MEG can be used to detect modulation of beta oscillations with changing attention. In our study, the sensory stimulus generated a large (~ 100%) drop in beta band amplitude post-stimulation (measured as a signal change relative to baseline standard deviation). This is typical of the types of signals that have been measured in OPM-MEG demonstrations to date, which most often comprise either evoked responses^32,77^ or induced oscillatory modulation^31,37^ elicited by simple paradigms. More importantly, our study also measured attentional modulation of the beta signal; this generated a ~ 20% change in amplitude during the attentional cue window, for the left and right somatosensory cortices respectively (see Fig. 3). The fact that this much more subtle modulation, which has been observed previously using conventional MEG measurements^48^can be reliably measured using OPMs provides exciting evidence for the high-fidelity metrics that are now afforded by OPM-MEG systems.

The scanner itself warrants discussion. We employed triaxial OPM sensors^78^ in an array design that has been well characterised by previous papers^37,50,51,79^. This design has significant advantages (over single or dual-axis OPM variants^33^) including better differentiation between signals originating inside and outside the brain^61,79^more homogeneous coverage of the cortex^50^ and larger overall signal capture^41^. However it does come at the cost of a slightly higher sensor noise floor^50^. In addition, we employed a background field control^51,54^ based on bi-planar coil technology^46,47^ that enabled viable OPM operation in the presence of subject movement. Consequently, our subjects were not constrained to a helmet as would be the case for conventional MEG. However, a limitation in this study was that the experimental nature of the system meant that not all sensors were available for every scan, and consequently the channel count was different for different subjects. To mitigate this, we always tried to populate the sensor slots in the helmet to provide good coverage of the sensorimotor cortices, minimising the impact of sensor count changes. In addition, because we contrasted conditions within the same experiment (rather than conditions between subject groups) the effect of changing sensor count between subjects should be minimised. Nevertheless, in a subset of subjects, coverage was compromised, and this would make some types of analysis challenging – for analysis the measurement of whole brain functional connectivity.

From a neuroscientific point of view, the observation of decreased beta amplitude during the presentation of attended (relative to unattended) stimuli has been observed previously, and supports the notion that the beta rhythm carries a top-down influence which helps to inhibit primary cortical areas. Interestingly, the most pronounced effects were during the attentional cue—i.e. in a window where no direct sensory stimulation was taking place. In this window, a cue directing attention to the ipsilateral hand caused no significant change in beta amplitude from baseline. However, a cue directing attention to the contralateral hand saw a significant drop in beta amplitude, making it tempting to speculate that role of this drop is to release top-down inhibition and enhance excitability in the brain region where the sensory stimulus is to be processed. In addition to the attentional cue, beta band amplitude was also significantly diminished during presentation of attended compared to non-attended stimuli—an effect most prominent before and after, but not during tactile stimulation (where it is likely masked by the sensory response itself). Again these differences likely reflect the release of top-down inhibition on the primary sensory cortices.

Our results also show that the observed beta effects are driven (in part at least) by bursts of electrophysiological activity. Our HMM detects the recurrence of states in which a brain region enters into a period of activity with a specific spectro-temporal profile^76^. Previous work suggests that the state whose timecourse correlates most with the beta envelope delineates “beta-bursts”^10,73^. Given the methodology, the fact that bursts are less likely during periods of lower beta amplitude is not surprising. However, it is noteworthy that a period of high beta could result from either a higher burst amplitude or a change in the probability of a burst occurring (independent of its amplitude). Here our results (Fig. 5) show significant modulation of the latter (burst probability) with attention (independent of amplitude); i.e. bursts are less likely to occur in right sensorimotor cortex when attending to the left hand, compared to the right hand (and vice versa). Our follow up analysis supports this; for example Fig. 6 shows a trend that the number of bursts was lower, and the duration of those bursts shorter, during periods of attention compared to inattention. The effect of attention on burst amplitude was less clear, with a trend towards higher amplitude bursts during inattention in the left hemisphere but no measurable effect in the right hemisphere. Taken together these results suggest that switching sensory attention has the effect of modulating both the number of bursts in primary sensory areas, but also the burst characteristics.

A notable finding is that bursts are not solely attributed to electrophysiological fluctuations in the beta band; indeed in agreement with previous work^10^. Figure 4B shows a pan-spectral profile for the burst state, indicating contributions from a range of frequencies. Specifically, there is a clear peak in the alpha range and a marked “shoulder” in the beta range observable in the “beta burst” state in both the left and right primary sensory cortices. The precise nature of this pan-spectral effect is unclear from the current analysis methods; it could be that there are separate (independent) effects in both the alpha and beta ranges within a single a single burst; alternatively it is possible that the “beta” frequencies identified could result from aperiodic components of lower frequency (e.g. alpha) activity. Previous work ^e.g. 80^ has attempted to disentangle these effects, via identification of bursts of beta activity that don’t coincide with similar bursts in alpha. Further work ^e.g. 81^ has suggested that the precise amplitude/frequency of beta modulation, and more intriguingly the distinct Spectro-temporal profile of individual bursts, influences measurable behavioural traits (e.g. reaction times). Although our current methodology prevents such delineation, it would be interesting to ascertain whether the bursts that modulate with attention have spectro-temporal properties that are distinct from, for example, those that modulate with simple tactile stimulation. This is a topic for future work.

The current study has some limitations which should be mentioned. Firstly, to avoid confounds of a button press we eliminated both the target probe stimuli, and stimuli where subjects incorrectly pressed a button from our final analyses. This meant an imbalance in attended and unattended probe stimuli and for our final analysis we had to remove (randomly) a selection of non-attended stimuli. This meant that a proportion of the data were lost in our analysis. Secondly, while we removed epochs containing button presses, we acknowledge that motor preparation processes could still influence our results. Beta oscillations in sensorimotor cortex are well-established markers of both motor preparation and execution. Although our task was designed to isolate attentional processes, participants’ anticipation of potential motor responses could contribute to the observed beta modulations. A third limitation of the study design relates to the task timing. The beta response to somatosensory stimulation is traditionally characterised by a drop in beta amplitude during stimulation followed by a rebound (above baseline) on stimulus cessation^2^. A good deal of evidence suggests that these two responses (at least following movement) have different neural generators—e.g. with localisations typically placing the rebound anterior in the brain compared to the beta decrease. It would be informative to know how attention modulates both the decrease and rebound independently, however here we only probed beta decrease relative to the resting blocks and the probe stimuli were presented so rapidly that the rebound was never allowed to evolve. Future studies should take this into account. Finally, here we used an HMM to identify bursting; this differs from the traditionally used methods which involve frequency filtering to the beta band, computation of the instantaneous beta envelope, and thresholding to find periods of high amplitude. In principle the HMM provides a more principled approach, since the burst count, and burst durations are not defined by an arbitrary threshold applied to the envelope. Nevertheless, the HMM burst identification is affected by e.g. the mutual exclusivity and Markovian properties inherent to the model, and this should be considered when placing the present finding in the context of the current literature on bursts.

## Conclusion

Using OPM-MEG we have successfully measured attentional modulation of beta oscillations in somatosensory cortex. We found significantly decreased beta amplitude during presentation of attended compared to non-attended stimuli. This is in agreement with previous findings from conventional-MEG and supports the notion that beta oscillations mediate top-down inhibition. Moreover, our analyses showed that attention has a similar effect on the occurrence of pan-spectral bursts; specifically, we measured significant reduction in the probability of burst occurrence, and the number of bursts per unit time, for attended compared to non-attended stimuli. In sum, our results provide evidence that attentional modulation of beta oscillations is driven by changes in pan-spectral burst occurrence and add weight to the argument that OPM-MEG could become the technique of choice for non-invasive electrophysiological measurements.

## Electronic supplementary material

Below is the link to the electronic supplementary material.

Supplementary Material 1

## Data Availability

All data and code available on request to the authors (Braille paradigm repository, https://github.com/GonReina/Braille_paradigm.git). Upon publication, data will be made available on Zenodo alongside other data from our group.
