# Supplementary material for: OPM-MEG reveals dynamics of beta bursts underlying attentional processes in sensory cortex
Source: Sci Rep. 2025 Aug 19;15:30471. doi: 10.1038/s41598-025-08037-8 (PMC12365038; doi:10.1038/s41598-025-08037-8)
Supplement: Supplementary file 1 — Supplementary Material 1 [file 41598_2025_8037_MOESM1_ESM.docx]

**Appendix 1: Array formation variations**

As explained in our manuscript, the number of sensors varied for different subjects. To assess the potential impact of this, we performed an investigation into the brain coverage provided by both arrays. Using a forward solution based on the dipole and single shell models, we computed the Frobenius norm of the forward fields generated by all AAL derived source positions. These values were derived for all subjects (using the sensor locations/orientations that were determined in the experimental sessions). For each subject these values were normalised by their maximum value (across all AAL regions), averaged over subjects scanned with different sensor counts, and plotted for all brain regions. Results are shown in Figure A1. Differences in coverage were found to be minimal in regions of interest to this study (i.e. sensorimotor cortices). This allows us to derive results from these regions without introducing biases in source space outcomes resulting from coverage variations.


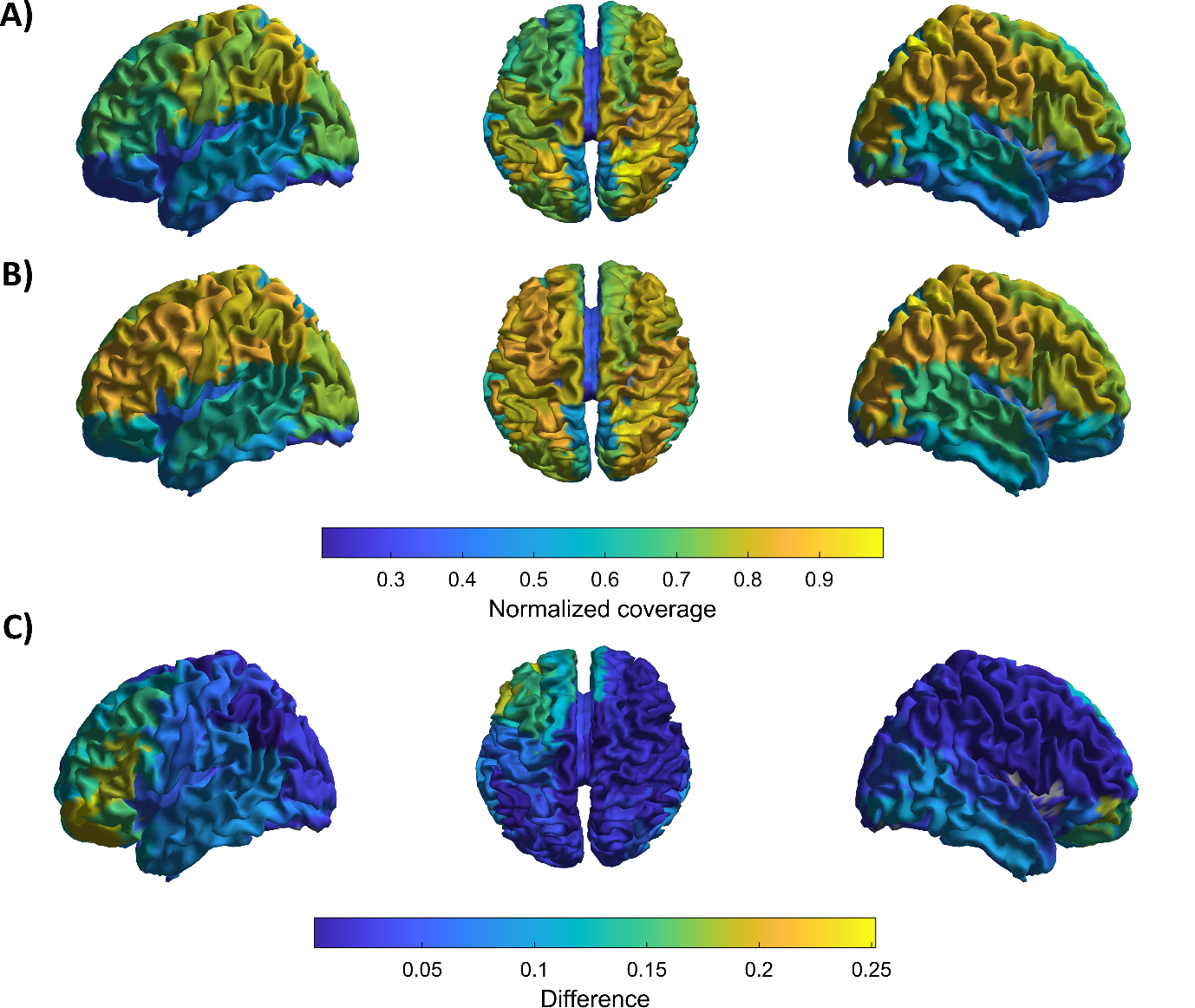


***Figure A1. Sensor brain coverage.*** *Brain surface colourmaps representing the normalized Frobenius norm of the AAL atlas regions for scans with A) 132 channels, B) 192 triaxial sensors. C) shows the difference between the two channel counts. The brain surface colourmaps were produced using the AAL atlas brain template in MATLAB (Version* [*R2023a*](Version%20R2022a.%20Natick,%20Massachusetts:%20The%20MathWorks%20Inc.)*, MathWorks Inc.).*
